# HMGB1 May Be a Biomarker for Predicting the Outcome in Patients with Polymyositis /Dermatomyositis with Interstitial Lung Disease

**DOI:** 10.1371/journal.pone.0161436

**Published:** 2016-08-18

**Authors:** Xiaoming Shu, Qinglin Peng, Xin Lu, Guochun Wang

**Affiliations:** Derpartment of Rheumatology, China-Japan Friendship Hospital, YingHua East Road, Chaoyang District, Beijing, 100029, China; Imperial College London, UNITED KINGDOM

## Abstract

**Objective:**

To investigate the significance of high mobility group box 1 (HMGB1) levels in polymyositis (PM) and dermatomyositis (DM) patients with interstitial lung disease and whether HMGB1 levels could predict disease outcome.

**Methods:**

HMGB1 levels were measured in sera from 34 patients with PM/DM and from 34 healthy controls by ELISA.

**Results:**

Significantly higher serum levels of HMGB1 were found in patients with PM [12.75 ng/ml (4.34–25.07 ng/ml), p < 0.001] and DM [20.75 ng/ml (3.80–124.88 ng/ml), p < 0.001] than in healthy controls [5.64 ng/ml (2.71–8.71 ng/ml)]. Importantly, the average HMGB1 level in PM/DM patients with interstitial lung disease (ILD) was 25.84 ng/ml, which is significantly higher than that in PM/DM patients without ILD [12.68 ng/ml] (p < 0.05). A receiver operating characteristic (ROC) curve analysis revealed that the serum HMGB1 cutoff value that best discriminated PM/DM patients with ILD from those without ILD was 14.5ng/ml. The area under the curve was 0.87±0.05, and the 95% Confidence interval (CI) was 0.77–0.98. The diagnostic sensitivity and specificity of this serum HMGB1 cutoff level was 84.6% and 89% respectively. Patients with higher levels of HMGB1 expression had lower overall survival rates and disease-free survival rates, whereas patients with lower levels of HMGB1 expression had higher survival rates.

**Conclusion:**

Multivariate analysis showed that HMGB1 expression is a prognostic indicator for patient survival. These data support the notion that HMGB1 overexpression is involved in PM/DM progression for patients with ILD and is relative to its poor clinical outcomes.

## Introduction

Polymyositis (PM) and Dermatomyositis (DM) are heterogeneous complex autoimmune diseases that primarily target skeletal muscles and skin. They are characterized by progressive systematic proximal muscle weakness and erythematous rash. PM/DM can also present with multi-organ or systemic manifestations and a significant increase in the incidence of interstitial lung disease (ILD) [1, 2]. Although the etiopathogenesis of PM/DM remains unclear, the hallmark of the disease is lymphocytic infiltration of muscles, muscle inflammation, and chronic muscle dystrophy [3, 4]. The prevailing paradigm is that muscle lesions in patients with PM/DM are populated with CD4^+^ T and CD8^+^ T lymphocytes and MHC-I, which orchestrate muscular damage [5–10]. At present, we are unable to make accurate predictions on the basis of clinical characteristics. Few reliable prognostic indicators were available.

Recently, it was found that HMGB1 protein is belong to the family of damage-associated molecular patterns, which includes endogenous ligands of pattern recognition receptors. HMGB1 could be released by necrotic cells and play a vital role in innate and adaptive immunity [11–13]. Increased serum and/or plasma levels of HMGB1 have been reported in several human autoimmune diseases such as systemic lupus erythematosus (SLE) and rheumatoid arthritis [13–15], indicating the involvement of HMGB1 in the pathogenesis of these autoimmune diseases. Although it has also been reported that HMGB1 may play an important role in the occurrence of PM/DM [16], the role of HMGB1 in the diagnosis of PM/DM patients who have ILD complications and how HMGB1 may help predict disease outcome remains poorly understood.

In this study, we analyzed the HMGB1 expression in serum samples from patients with new-onset PM/DM and compared these results to their clinical characteristics of PM/DM patients. We hope to identify new biomarkers or predicator of inflammatory myopathies for disease activity and progression of ILD and prognosis. Our results provide evidence that HMGB1 is over-expressed in new-onset PM/DM patients with ILD, and may serve as an indicator of disease progression and help predict poor outcomes.

## Materials and Methods

### Subjects

For this study, 34 Han Chinese patients with new-onset PM/DM who did not receive previous treatment were chosen from a cohort of 210 PM/DM patients. All study participants gave informed written consent for their participation. They consisted of 11 PM and 23 DM patients, 8 males and 26 females. Normal HMGB1 levels also were measured in 34 healthy control subjects, all of whom gave informed written consent for participation in this study. These individuals, who had no signs of inflammation, neoplasm, autoimmune and metabolic diseases, underwent a routine evaluation at the China-Japan Friendship Hospital Health Examination Department. There were no significant differences between the PM/DM patients and control subjects with regard to age or sex distribution. Inclusion in the present study required the new-onset patients as well as availability of appropriate clinical data. This study protocol was approved by the ethical committee of China-Japan Friendship Hospital.

### Reagents and antibodies

A commercial ELISA (enzyme-linked immunosorbent assay) kit specific for detecting serum concentrations of HMGB1 was purchased from SHINO-TEST (Kanagawa, Japan).

### Measurement of serum HMGB1 concentration

Serum samples were obtained from 34 patients at the time of their initial diagnosis, prior to any treatment. All blood samples were separated at 4°C within 60 min after venipuncture and collected in Sterile Eppendorf tubes (size: 1.0 ml). Then, it was stored at -80°C until analysis. Serum HMGB1 levels were measured using a commercially available ELISA kit for HMGB1 antigen according to the manufacturer’s protocol. Each sample was tested in duplicate. The 34 healthy sera were run in the same assay. The detection limit of this assay was 1.0 ng/ml.

### Clinical assessment

Complete medical records were obtained, and physical examinations and laboratory tests were conducted for all patients at their first visit. Routine laboratory assessments were performed, including determining of levels of serum creatine kinase (CK), aspartate aminotransferase (AST), lactate dehydrogenase (LDH), hydroxybutyrate dehydrogenase (HBDH), erythrocyte sedimentation rate (ESR), C-reactive protein (CRP), electromyography (EMG); and high resolution computerized tomography (HRCT).

### Statistical analysis

Data are presented as medians and interquartile ranges (25th–75th percentiles; IQRs), absolute values and percentages, or means ± a standard deviation (SD). The nonparametric data of survivors and non-survivors were compared using the Mann—Whitney U test, and categorical variables were compared using the chi-square test. To determine the prognostic accuracy of HMGB1 at initial visit, the receiver operating characteristic (ROC) curves were constructed and the areas under the curve (AUC) of the 95% confidence interval (CI) were calculated. The level of p values less than 0.05 were considered statistically significant for all tests. The analyses were performed using SPSS 15.0 software (SPSS, Chicago, IL, USA).

Non-parametric methods were used for statistical comparisons because the data showed a non-normal distribution. Statistical differences with respect to HMGB1 levels between independent groups were calculated using the Kruskall—Wallis test followed by the Mann—Whitney U test. The Wilcoxon signed rank test for paired samples was used to compare differences between variables in matched samples. Correlations between different variables in patients were identified using the Spearman rank correlation test. Fisher's exact probability test was used to assess differences between groups with regard to disease characteristics. Survival curves were plotted using the Kaplan—Meier method and compared using the log-rank test. The Cox proportional hazards model was used in a multivariate analysis to analyze the impact of several variables on survival. The following prognostic factors for mortality were analyzed: age at initial visit; sex; presence of skin ulcers, presence of typical rashes; ILD complications; cardiac involvement, levels of creatine kinase at initial visit. There were no missing values among the prognostic factors in the data set. All HMGB1 values are expressed as medians and as means ± standard error of the mean. Patients' age and disease duration are reported as the mean ± standard deviation. P values less than 0.05 were considered significant.

## Results

### Demographic characteristics, clinical and laboratory data of patients and controls

The clinical parameters of all patients are summarized in Table 1, which include age, gender, and laboratory data. We found that there were no significant differences between the ages, gender, creatine kinase levels, and ESR in PM/DM patients with and without ILD.

**Table 1:** Clinical profile of PM/DM patients.

	All	PM	DM	PM/DM-ILD	PM/DM without ILD	P value
No. of patients	34	11	23	13	21	NA
Male: Female ratio	1:3.4	1:3.5	1:2.1	1:2.3	1:2.7	>0.05
Mean age ±S.D at initial visit (y)	47.8±15.4	45±18.3	49.5±13.6	47.3±16.2	46.3±19.4	>0.05
Creatine kinase at initial visit, mean ±SD (IU/L)	1391.6±956	2300.4±741	895.9±288	1218±455	1631±461	>0.05
ESR at initial visit, mean ±SD (mm/h)	24.7±2.5	25.4±3.7	24.3±3.4	24.6±3.2	25.7±3.8	>0.05

PM: polymyositis, DM: dermatomyositis, ILD: interstitial lung disease, PM/DM -ILD: myositis complicated with interstitial lung disease; ESR: erythrocyte sedimentation rate; SD: standard deviation. NA: not assessed.

### Increased serum HMGB1 expression in PM/DM patients

**Fig 1:**
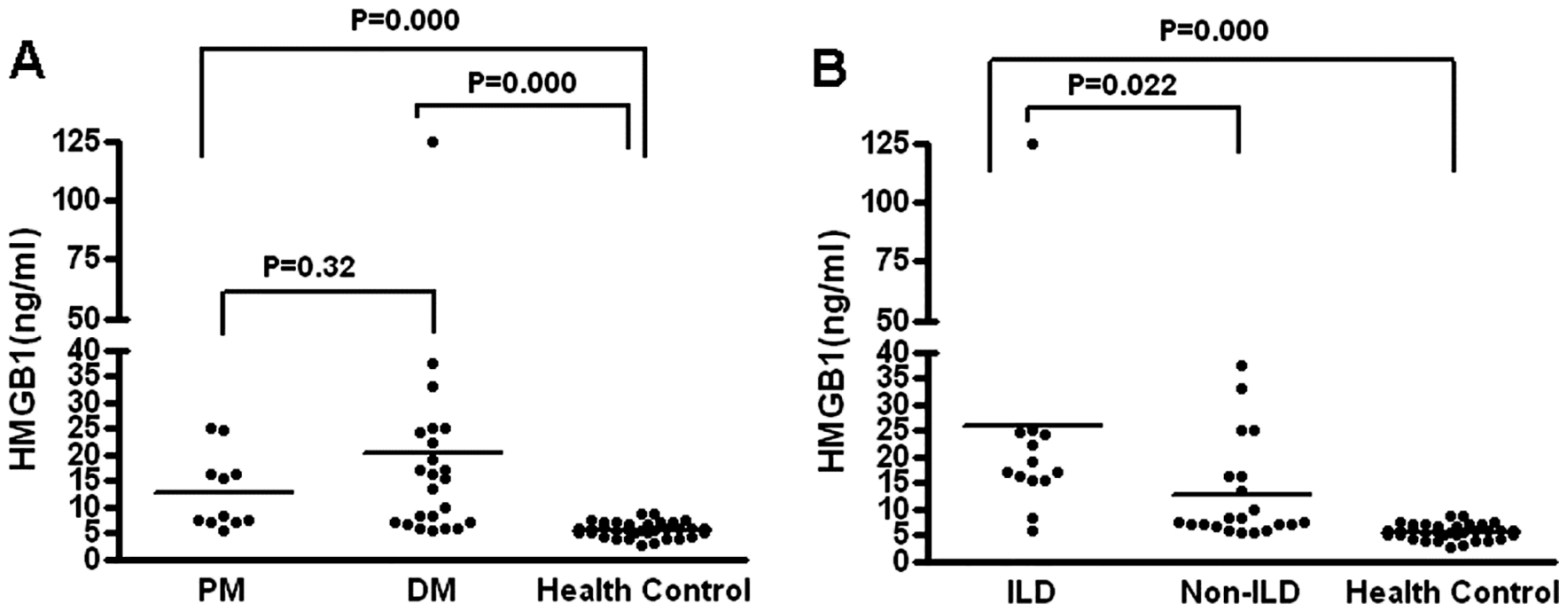
Serum HMGB1 levels in PM/DM patients with and without ILD. A) Significantly higher serum levels of HMGB1 were found in PM patients [12.75 ng/ml (4.34–25.07 ng/ml), p <0.001], and in DM patients [20.75ng/ml (3.80–124.88 ng/ml), P<0.001] than in healthy controls [5.64 ng/ml (2.71–8.71 ng/ml)]. B) Importantly, HMGB1 levels in PM/DM patients with interstitial lung disease (ILD) were 25.84 ng/ml, which was significantly higher than those in PM/DM patients without ILD [12.68ng/ml] (P<0.05).

To assess the relationship between HMGB1 expression and PM/DM disease progression, we measured serum HMGB1 levels in PM/DM patients with and without ILD by using ELISA. The results are shown in Fig 1. Serum levels of HMGB1 in patients with PM were 12.75 ng/ml (4.34–25.07 ng/ml), and those in DM were 20.75 ng/ml (3.80–124.88 ng/ml), while those in healthy controls were 5.64 ng/ml (2.71–8.71 ng/ml). HMGB1 levels in both PM or DM patients were significantly higher than those healthy controls. However, there were no significant differences between the HMGB1 levels of PM and DM patients (Fig 1A). In contrast, the levels of HMGB1 in PM/DM patients with ILD were markedly higher than those in patients without ILD (Fig 1B). These results indicate that HMGB1 levels can be a clinical marker for distinguish PM/DM patients with ILD from those without ILD.

### Diagnostic value of HMGB1 in PM/DM patients with ILD

**Fig 2:**
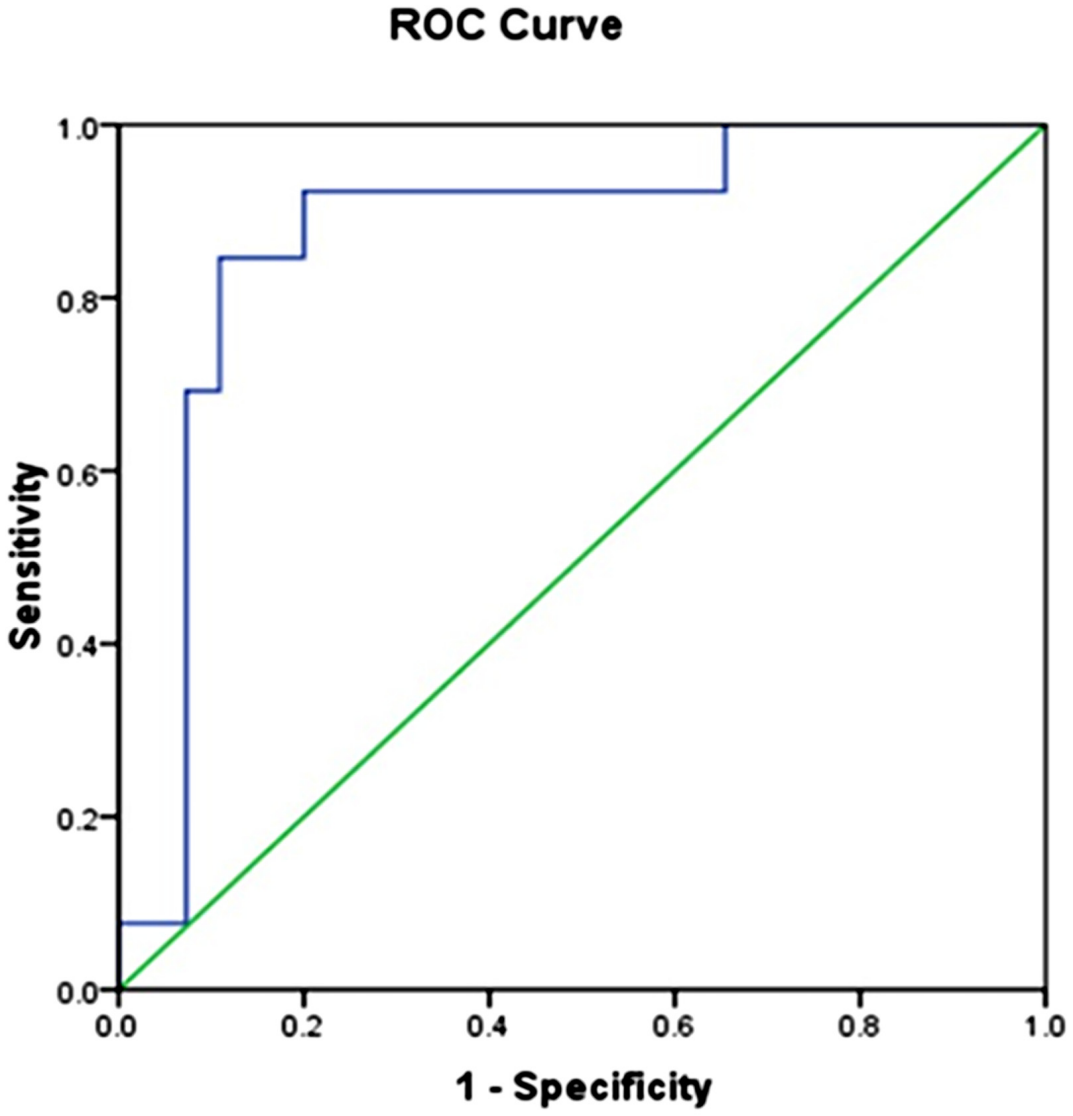
A receiver operating characteristic (ROC) curve analysis to determine an HMGB1 cutoff value (14.5ng/ml), which best distinguishes PM/DM patients with ILD from those without ILD. The area under curve (AUC) is 0.87±0.05, and the 95% CI is 0.77–0.98. The diagnostic sensitivity and specificity were 84.6% and 89%, respectively.

To explore the exact impact of HMGB1 levels on the disease progression of PM/DM patients with ILD, we examined the ROC curves for these patients. The results are shown in Fig 2. The ROC cutoff value of serum HMGB1 levels that distinguishes PM/DM patients with ILD from those without ILD is 14.5ng/ml. The range value of AUC values for PM/DM patients with and without ILD is 0.77–0.98. The diagnostic sensitivity and specificity for PM/DM with ILD is 84.6%and 89% respectively. These data demonstrated that there is a significant difference between the HMGB1 levels of PM/DM patients with ILD and the HMGB1 levels in those without ILD. This finding suggested that serum HMGB1 levels could be regarded as parameter for distinguishing these two patient populations.

### HMGB1 expression correlates with prognosis in PM/DM patients

**Fig 3:**
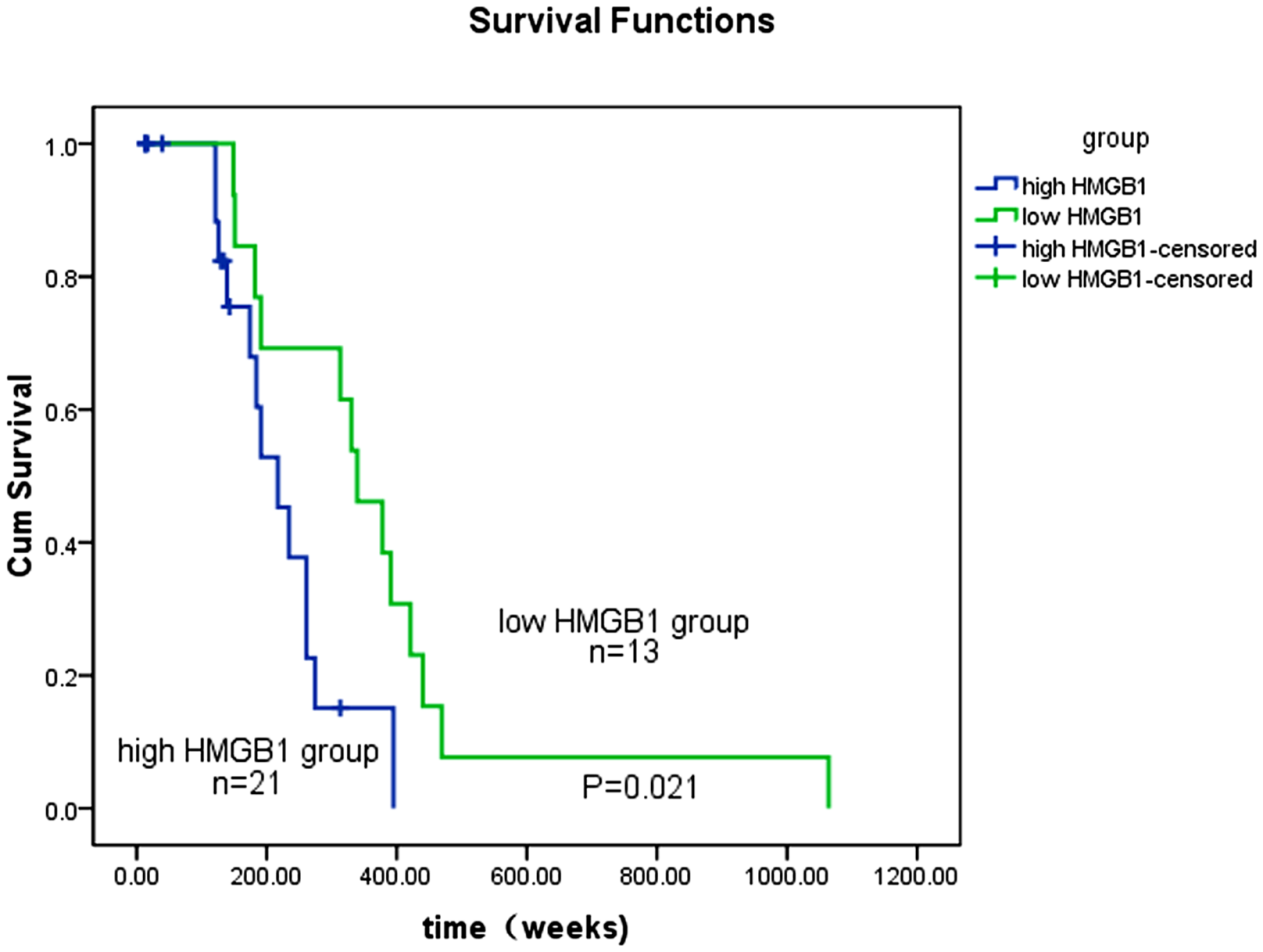
Kaplan—Meier survival analysis of overall survival rates in all patients according to serum HMGB1 levels. The log-rank test was used to calculate p values. High HMGB1 levels were ≥14.5 ng/ml, while low HMGB1 levels were <14.5ng/ml.

The impact of serum HMGB1 levels on long-term survival rates also was investigated as well. We analyzed overall survival times for patients with different HMGB1 levels. The results are shown in Fig 3. Patients with higher HMGB1 (≥ 14.5 ng/ml, ROC cutoff value) had significantly worse prognosis than those with lower HMGB1 levels (< 14.5 ng/ml) (log-rank test, p = 0.021) in a 1200-week survival analysis. These findings also indicate that serum HMGB1 levels are a strong prognostic factor for PM/DM patient survival.

Multivariate Cox hazard analysis also confirms that serum HMGB1 higher levels in PM/DM patients (≥ 14.5 ng/ml, ROC cutoff value) (HR = 2.10; 95% CI 1.13–5.52; p = 0.023) are an adverse prognostic factor. In addition to high HMGB1 levels, ILD complication was also associated with poorer prognosis (HR = 2.52; 95% CI 1.76–6.03; p = 0.021) (Table 2). No significant differences were found regarding age, gender, presence of typical rashes, presence of skin ulcers, or cardiac involvement.

**Table 2:** Multivariate Cox Proportional Hazard Model Evaluation of Mortality.

	Hazard Ratio	Confidence Interval	P Value
Age at initial visit (years)	1.02	0.98–1.05	0.053
Sex	0.75	0.18–2.23	0.371
Typical rashes	1.01	1.00–1.02	0.065
Skin ulcers	1.57	0.63–3.90	0.059
Creatine kinase at initial visit	1.07	0.33–3.49	0.704
Complicated with ILD	2.52	1.76–6.03	0.021*
Cardiac involvement	0.51	0.12–1.04	0.447
HMGB1 level (cutoff 14.5ng/ml)	2.10	1.13–5.52	0.023*

*<0.05

## Discussion

In the present study, we retrospectively investigated serum HMGB1 levels of total 34 PM/DM patients with and without ILD, especially their association with clinical features and potential prognostic value. Our results indicate that serum HMGB1 levels in PM/DM patients are an important biomarker associated with their clinical characteristics as well as with disease outcome.

HMGB1 is a multifunctional protein that is involved in transcription control, DNA repair and response to infection and inflammation. HMGB1 is rapidly moved from the nucleus into the cytoplasm and circulation during inflammation [16]. It also promotes the release of cytokines [11, 12] and attracts inflammatory cells [17]. It was reported that HMGB1 is involved in a few autoimmune diseases such as RA [13], SLE [14] and idiopathic inflammatory myopathies (IIM) [15]. One possible mechanism for HMGB1’s role in autoimmune diseases is that it may induce muscular dysfunction in PM /DM patients [18]. It also promotes autophagy of muscle fibers [19]. An experiment showed that HMGB1 proteins exist in adult skeletal muscle fibers [20]. These studies demonstrated that HMGB1 causes muscle weakness by triggering the dysfunction of muscle fibers. Therefore, HMGB1 levels may be related to the clinical characteristic of PM/DM patients. Our results showed that HMGB1 levels in PM/DM patients are significantly higher than those in healthy controls. However, there is no difference between the HMGB1 levels of PM and DM patients. Furthermore, the PM/DM -ILD patients have markedly higher levels of HMGB1 than do PM/DM patients without ILD. It is well known that PM/DM involved the lungs in addition to affecting muscle and skin [21]. Clinical studies shown that PM/DM patients with ILD have bad disease-free survival time [22–24]. The present data indicates that the HMGB1 gene also be involved in the pathogenesis of ILD.

PM/DM is a kind of autoimmune disease in which immunological molecules attack skeletal muscules [25]. Emerging evidence is showing that HMGB1 promotes the release of proinflammatory cytokines and regulates muscle fiber regeneration [11, 15, 26]. It appears to be a late mediator in inflammation [17, 27, 28]. Clinical investigations have shown that the presence of autoantibodies to HMGB1 are beneficial to sepsis patients [29]. Our ROC curves show that high HMGB1 levels differentiate PM/DM patients with ILD from those without ILD (AUC = 0.87±0.05; 95% CI = 0.77–0.98). Our data revealed that patients with high levels of HMGB1 had short disease-free survival time compared with patients with lower levels of HMGB1 expression.

The prognostic factors of PM/DM-ILD patients have been extensively studied, which include acute progressive ILD, auto-antibodies to different self-antigen high levels of serum ferritin [30–33] and myocardial infarction [34, 35]. Interest, a high HMGB levels occurred in PM/DM and myocardial infarction. This demonstrated that HMGB1 may have effect on muscle function. Our Kaplan—Meier survival curves and Cox hazard analyses indicate that serum HMGB1 levels in the PM/DM-ILD group is markedly higher than that in the non-ILD PM/DM group. Moreover, patients in the PM/DM—ILD group had poorer survival rates than did the non-ILD PM/DM group. Our results only indicated that a high serum HMGB1 may predict the outcomes in PM/DM with ILD. More underlying mechanism need be clarified.

## Conclusion

Our multivariate analysis results revealed that HMGB1 expression is an important indicator of disease progression in PM/DM patients. It also may be a useful marker for distinguishing PM/DM patients with ILD from those without IID and a possible prognostic indicator for patient survival.

## References

[1] Bohan A, Peter JB. Polymyositis and dermatomyositis (second of two parts). N Engl J Med. 1975;292(8):403–7. 10.1056/NEJM197502202920807 . 1089199

[2] Dalakas MC, Hohlfeld R. Polymyositis and dermatomyositis. Lancet. 2003;362(9388):971–82. 10.1016/S0140-6736(03)14368-1 . 14511932

[3] Emslie-Smith AM, Engel AG. Microvascular changes in early and advanced dermatomyositis: a quantitative study. Ann Neurol. 1990;27(4):343–56. 10.1002/ana.410270402 . 2353792

[4] Kissel JT, Halterman RK, Rammohan KW, Mendell JR. The relationship of complement-mediated microvasculopathy to the histologic features and clinical duration of disease in dermatomyositis. Arch Neurol. 1991;48(1):26–30. . 1986724 10.1001/archneur.1991.00530130034016

[5] Arahata K, Engel AG. Monoclonal antibody analysis of mononuclear cells in myopathies. III: Immunoelectron microscopy aspects of cell-mediated muscle fiber injury. Ann Neurol. 1986;19(2):112–25. 10.1002/ana.410190203 . 3008636

[6] Dalakas MC. Polymyositis, dermatomyositis and inclusion-body myositis. N Engl J Med. 1991;325(21):1487–98. 10.1056/NEJM199111213252107 . 1658649

[7] Sundaram C, Uppin MS, Meena AK. Major histocompatibility complex class I expression can be used as a diagnostic tool to differentiate idiopathic inflammatory myopathies from dystrophies. Neurol India. 2008;56(3):363–7. . 18974565 10.4103/0028-3886.43457

[8] Vitadello M, Doria A, Tarricone E, Ghirardello A, Gorza L. Myofiber stress-response in myositis: parallel investigations on patients and experimental animal models of muscle regeneration and systemic inflammation. Arthritis Res Ther. 2010;12(2):R52 10.1186/ar2963 20334640 PMC2888201

[9] Li CK, Varsani H, Holton JL, Gao B, Woo P, Wedderburn LR, et al MHC Class I overexpression on muscles in early juvenile dermatomyositis. J Rheumatol. 2004;31(3):605–9. . 14994413

[10] Chauhan AK, Moore TL, Bi Y, Chen C. FcgammaRIIIa-Syk Co-signal Modulates CD4+ T-cell Response and Up-regulate TLR expression. J Biol Chem. 2015. 10.1074/jbc.M115.684795 PMC4714221 26582197

[11] Harris HE, Andersson U, Pisetsky DS. HMGB1: a multifunctional alarmin driving autoimmune and inflammatory disease. Nat Rev Rheumatol. 2012;8(4):195–202. 10.1038/nrrheum.2011.222 . 22293756

[12] Lotze MT, Tracey KJ. High-mobility group box 1 protein (HMGB1): nuclear weapon in the immune arsenal. Nat Rev Immunol. 2005;5(4):331–42. 10.1038/nri1594 . 15803152

[13] Pisetsky DS, Erlandsson-Harris H, Andersson U. High-mobility group box protein 1 (HMGB1): an alarmin mediating the pathogenesis of rheumatic disease. Arthritis Res Ther. 2008;10(3):209 10.1186/ar2440 18598385 PMC2483460

[14] Abdulahad DA, Westra J, Bijzet J, Limburg PC, Kallenberg CG, Bijl M. High mobility group box 1 (HMGB1) and anti-HMGB1 antibodies and their relation to disease characteristics in systemic lupus erythematosus. Arthritis Res Ther. 2011;13(3):R71 10.1186/ar3332 21548924 PMC3218880

[15] Grundtman C, Bruton J, Yamada T, Ostberg T, Pisetsky DS, Harris HE, et al Effects of HMGB1 on in vitro responses of isolated muscle fibers and functional aspects in skeletal muscles of idiopathic inflammatory myopathies. FASEB J. 2010;24(2):570–8. 10.1096/fj.09-144782 . 19837864

[16] Muller S, Scaffidi P, Degryse B, Bonaldi T, Ronfani L, Agresti A, et al New EMBO members' review: the double life of HMGB1 chromatin protein: architectural factor and extracellular signal. EMBO J. 2001;20(16):4337–40. 10.1093/emboj/20.16.4337 11500360 PMC125571

[17] Andersson U, Tracey KJ. HMGB1 is a therapeutic target for sterile inflammation and infection. Annu Rev Immunol. 2011;29:139–62. 10.1146/annurev-immunol-030409-101323 21219181 PMC4536551

[18] Zong M, Bruton JD, Grundtman C, Yang H, Li JH, Alexanderson H, et al TLR4 as receptor for HMGB1 induced muscle dysfunction in myositis. Ann Rheum Dis. 2013;72(8):1390–9. 10.1136/annrheumdis-2012-202207 . 23148306

[19] Kang R, Livesey KM, Zeh HJ 3rd, Lotze MT, Tang D. HMGB1 as an autophagy sensor in oxidative stress. Autophagy. 2011;7(8):904–6. . 21487246 10.4161/auto.7.8.15704

[20] Cseri K, Vincze J, Cseri J, Fodor J, Csernatony Z, Csernoch L, et al HMGB1 expression and muscle regeneration in idiopathic inflammatory myopathies and degenerative joint diseases. J Muscle Res Cell Motil. 2015;36(3):255–62. 10.1007/s10974-015-9411-7 . 25761565

[21] Ichikado K, Johkoh T, Ikezoe J, Takeuchi N, Kohno N, Arisawa J, et al Acute interstitial pneumonia: high-resolution CT findings correlated with pathology. AJR Am J Roentgenol. 1997;168(2):333–8. 10.2214/ajr.168.2.9016201 . 9016201

[22] Marie I, Hachulla E, Cherin P, Dominique S, Hatron PY, Hellot MF, et al Interstitial lung disease in polymyositis and dermatomyositis. Arthritis Rheum. 2002;47(6):614–22. 10.1002/art.10794 . 12522835

[23] Marie I, Hatron PY, Dominique S, Cherin P, Mouthon L, Menard JF. Short-term and long-term outcomes of interstitial lung disease in polymyositis and dermatomyositis: a series of 107 patients. Arthritis Rheum. 2011;63(11):3439–47. 10.1002/art.30513 . 21702020

[24] Won Huh J, Soon Kim D, Keun Lee C, Yoo B, Bum Seo J, Kitaichi M, et al Two distinct clinical types of interstitial lung disease associated with polymyositis-dermatomyositis. Respir Med. 2007;101(8):1761–9. 10.1016/j.rmed.2007.02.017 . 17428649

[25] Vincze M, Danko K. Idiopathic inflammatory myopathies. Best Pract Res Clin Rheumatol. 2012;26(1):25–45. 10.1016/j.berh.2012.01.013 . 22424191

[26] De Mori R, Straino S, Di Carlo A, Mangoni A, Pompilio G, Palumbo R, et al Multiple effects of high mobility group box protein 1 in skeletal muscle regeneration. Arterioscler Thromb Vasc Biol. 2007;27(11):2377–83. 10.1161/ATVBAHA.107.153429 . 17872450

[27] Wang H, Bloom O, Zhang M, Vishnubhakat JM, Ombrellino M, Che J, et al HMG-1 as a late mediator of endotoxin lethality in mice. Science. 1999;285(5425):248–51. Epub 1999/07/10. . 10398600 10.1126/science.285.5425.248

[28] Abeyama K, Stern DM, Ito Y, Kawahara K, Yoshimoto Y, Tanaka M, et al The N-terminal domain of thrombomodulin sequesters high-mobility group-B1 protein, a novel antiinflammatory mechanism. J Clin Invest. 2005;115(5):1267–74. 10.1172/JCI22782 15841214 PMC1077171

[29] Barnay-Verdier S, Fattoum L, Borde C, Kaveri S, Gibot S, Marechal V. Emergence of autoantibodies to HMGB1 is associated with survival in patients with septic shock. Intensive Care Med. 2011;37(6):957–62. Epub 2011/03/02. 10.1007/s00134-011-2192-6 . 21359606

[30] Hoshino K, Muro Y, Sugiura K, Tomita Y, Nakashima R, Mimori T. Anti-MDA5 and anti-TIF1-gamma antibodies have clinical significance for patients with dermatomyositis. Rheumatology (Oxford). 2010;49(9):1726–33. Epub 2010/05/27. 10.1093/rheumatology/keq153 . 20501546

[31] Nakashima R, Imura Y, Kobayashi S, Yukawa N, Yoshifuji H, Nojima T, et al The RIG-I-like receptor IFIH1/MDA5 is a dermatomyositis-specific autoantigen identified by the anti-CADM-140 antibody. Rheumatology (Oxford). 2010;49(3):433–40. Epub 2009/12/18. 10.1093/rheumatology/kep375 . 20015976

[32] Gono T, Kawaguchi Y, Satoh T, Kuwana M, Katsumata Y, Takagi K, et al Clinical manifestation and prognostic factor in anti-melanoma differentiation-associated gene 5 antibody-associated interstitial lung disease as a complication of dermatomyositis. Rheumatology (Oxford). 2010;49(9):1713–9. Epub 2010/05/26. 10.1093/rheumatology/keq149 . 20498012

[33] Tanizawa K, Handa T, Nakashima R, Kubo T, Hosono Y, Aihara K, et al The prognostic value of HRCT in myositis-associated interstitial lung disease. Respir Med. 2013;107(5):745–52. Epub 2013/03/15. 10.1016/j.rmed.2013.01.014 . 23485097

[34] Takahashi M. High-Mobility Group Box 1 Protein in Myocardial Infarction: Should it be Stimulated or Inhibited? J Atheroscler Thromb. 2015;22(6):553–4. 10.5551/jat.ED008 . 25752364

[35] Nakamura Y, Suzuki S, Shimizu T, Miyata M, Shishido T, Ikeda K, et al High Mobility Group Box 1 Promotes Angiogenesis from Bone Marrow-derived Endothelial Progenitor Cells after Myocardial Infarction. J Atheroscler Thromb. 2015;22(6):570–81. 10.5551/jat.27235 . 25735431

